# Interest in Supportive Services Among Black Working and Non-Working Caregivers in the Deep South

**DOI:** 10.1093/geroni/igaa057.2554

**Published:** 2020-12-16

**Authors:** Olivio Clay, Fayron Epps, Cathy Scott, Fawn Cothran, Ishan Williams

**Affiliations:** 1 University of Alabama at Birmingham, Birmingham, Alabama, United States; 2 Emory University, Atlanta, Georgia, United States; 3 University of Tennessee at Chattanooga, Chattanooga, Tennessee, United States; 4 UC Davis Betty Irene Moore School of Nursing, Sacramento, California, United States; 5 University of Virginia, Charlottesville, Virginia, United States

## Abstract

The current investigation provides information on supportive services caregivers said they would be interested in if they were made available. Participants were recruited from the Birmingham, AL metro area and received a $25 gift card for completing a telephone interview. Of the 38 caregivers enrolled, 18 (47.37%) reported being currently employed and working an average of 33.92 hours per week (range = 5 – 60). Participants were caring for individuals with average scores on the AD8 Dementia Screening Scale of 7.3 and 10.4 on the Clinical Dementia Scale Sum of Boxes (above the cutoffs for probable dementia). Thirty-three caregivers (87%) reported being interested in at least one of the services listed with differences observed for services that would be preferred by working vs. non-working caregivers. These data will be utilized to provide initial support for a multicomponent intervention that may be effective in reducing negative outcomes within Black caregivers.

